# Pregnancy-Related Mortality Due to Cardiovascular Conditions: Maternal Mortality Review Committees in 32 U.S. States, 2017 to 2019

**DOI:** 10.1016/j.jacadv.2024.101382

**Published:** 2024-11-08

**Authors:** Joan Briller, Susanna L. Trost, Ashley Busacker, Naima T. Joseph, Nicole L. Davis, Emily E. Petersen, David A. Goodman, Lisa M. Hollier

**Affiliations:** a Division of Cardiology, Department of Medicine, University of Illinois Chicago, Chicago, Illinois, USA; b Division of Reproductive Health, National Center for Chronic Disease Prevention and Health Promotion, Centers for Disease Control and Prevention, Atlanta, Georgia, USA; c Division of Maternal-Fetal Medicine, Department of Obstetrics and Gynecology, Boston Medical Center, Boston University School of Medicine, Boston, Massachusetts, USA; d United States Public Health Service, Commissioned Corps, Rockville, Maryland, USA

**Keywords:** cardiomyopathy, care coordination, knowledge, preventability, quality care, recommendations

## Abstract

**Background:**

Cardiomyopathy (CM) and other cardiovascular conditions (OCVs) are among the most frequent causes of pregnancy-related death in the United States.

**Objectives:**

The purpose of this paper was to report demographic and clinical characteristics, preventability, contributing factors, and Maternal Mortality Review Committee (MMRC) recommendations among pregnancy-related deaths with underlying causes of CM, OCVs, and the 2 combined (cardiovascular conditions, CV).

**Methods:**

We analyzed pregnancy-related death data from MMRCs in 32 states, occurring during 2017 to 2019, with MMRC-determined underlying causes of CVs. We describe distributions of demographic characteristics, present the most frequent contributing factor classes, and provide example MMRC prevention recommendations.

**Results:**

Among 210 pregnancy-related deaths due to CVs, 84 (40%) were due to CM and 126 (60%) to OCVs. More than half (51.2%) of CM deaths were among non-Hispanic Black persons. Two-thirds (66%) of all CV deaths occurred among people <35 years old. Approximately 53% of CM deaths and 31% of OCV deaths occurred 43 to 365 days postpartum. Over 75% of pregnancy-related deaths due to CVs were determined by MMRCs to be preventable. The 5 most frequent contributing factor classes accounted for 50% of the total MMRC-identified contributing factors. MMRC prevention recommendations occur at multiple levels.

**Conclusions:**

Most pregnancy-related deaths due to CM and OCV are preventable. Example MMRC recommendations provided in this report illustrate prevention opportunities that address contributing factors, including broader awareness of urgent warning signs, improved handoffs for care coordination and continuity, and expanded accessibility of community-based comprehensive and integrated care services.

The pregnancy-related mortality ratio in the United States has not improved over the last 20 years.^1^ Considerable racial-ethnic disparities in pregnancy-related mortality persist, with pregnancy-related mortality ratios among non-Hispanic Native Hawaiian or other Pacific Islander persons 4 times higher, non-Hispanic Black persons 3 times higher, and non-Hispanic American Indian or Alaska Native persons 2 times higher than among non-Hispanic White persons.^1^

Maternal Mortality Review Committees (MMRCs) provide a deep understanding of pregnancy-related mortality through detailed case reviews by a multidisciplinary group of clinical and nonclinical individuals.^2^ A recent report from MMRCs in 36 states identified differences in the leading underlying cause of pregnancy-related death by race-ethnicity.^3^ Overall, cardiac and coronary conditions (excluding cardiomyopathy [CM]) and CM were the second most frequent underlying cause of pregnancy-related deaths (20.6%) and among non-Hispanic Black persons, they were the most frequent underlying causes, accounting for 30% of pregnancy-related deaths.^3^ A prior analysis of cardiovascular deaths from a state-based MMRC found that the majority of deaths were due to acquired heart disease, with CM the most common etiology.^4^

The purpose of this analysis is to provide demographic and clinical information on specific cardiovascular causes of pregnancy-related deaths, to identify factors contributing to these deaths, and share example recommendations made by MMRCs to reduce preventable cardiovascular deaths among the most frequently identified contributors.

## Methods

### Study design and population

Using Maternal Mortality Review Information Application (MMRIA) data shared with the Centers for Disease Control and Prevention (CDC) by MMRCs, we analyzed data from the 32 states contributing data with pregnancy-related deaths with an MMRC-determined cause of CM and other cardiovascular conditions (OCVs) occurring among residents from 2017 to 2019 (Supplemental Appendix). In some states, only partial years of data were shared. We refer to the combination of CM and OCV as cardiovascular conditions (CV). This study did not involve human subjects as defined in 45 CFR 46.102(e) and therefore was not reviewed by an Institutional Review Board.

### Variables

Race and ethnicity were derived from the birth or fetal death record. If missing on the birth or fetal death record or if a linked birth or fetal death record was not available, race and ethnicity were derived from the death record and classified as Hispanic, non-Hispanic American Indian or Alaska Native, non-Hispanic Asian, non-Hispanic Black, non-Hispanic Native Hawaiian or other Pacific Islander, non-Hispanic White, or non-Hispanic another/multiple races using previously described methods.^3^ Age at death was based on the death record and categorized as ages 15 to 19 years, 20 to 24 years, 25 to 29 years, 30 to 34 years, 35 to 39 years, 40 to 44 years, and 45 years and older.

Methods for determining timing of death in relation to pregnancy have been described previously.^3^ Briefly, timing of death was assigned by using the number of days between the date of death and the end of pregnancy, as documented by the MMRC abstractor, or as calculated by using the number of days between the date of death on the death record and the date of birth or fetal death on the linked birth or fetal death record by CDC.^3^ If the specific number of days was missing, deaths that the MMRC abstractor classified as pregnant at the time of death, or with the standard pregnancy checkbox on the death certificate marked as pregnant at the time of death, were classified as during pregnancy.^3^

MMRCs determine a pregnancy-associated death (death during pregnancy or within 1 year of the end of pregnancy) to be pregnancy related if the death was from a pregnancy complication, a chain of events initiated by pregnancy, or the aggravation of an unrelated condition by the physiologic effects of pregnancy. The underlying cause of death is defined as the disease or injury which initiated the chain of events leading directly to death, or the circumstances of the accident or violence which produced the fatal injury. The MMRC-determined underlying cause of death 1) is coded using a standardized list of 20 major categories and 69 subcategories^3^^,^^5^ and 2) may vary from official underlying cause of death documented on the death record due to the multidisciplinary review and additional information available to the MMRC.

CM includes deaths attributed to postpartum/peripartum CM, hypertrophic CM, and other CM/not otherwise specified (NOS). OCV includes deaths attributed to coronary artery disease/myocardial infarction/atherosclerotic cardiovascular disease, pulmonary hypertension, acquired and congenital valvular heart disease, vascular aneurysm/dissection, hypertensive cardiovascular disease, Marfan syndrome, conduction defects/arrhythmias, vascular malformations outside the head and coronary arteries, and cardiovascular/NOS (such as congestive heart failure, cardiomegaly, cardiac hypertrophy, cardiac fibrosis, and nonacute myocarditis). In a previous publication,^3^ these deaths were titled “cardiac and coronary conditions.” Hypertensive disorders of pregnancy (including gestational hypertension, preeclampsia, and superimposed preeclampsia) are disorders unique to pregnancy and are categorized separately. Cerebrovascular accidents are also categorized separately by MMRCs, and neither are included in this analysis.

MMRCs also make determinations for circumstances surrounding each death, including whether obesity, substance use disorder, and mental health conditions other than substance use disorder were a circumstance of the death. In May 2020, an additional field was added to the MMRIA Committee Decisions Form to document the MMRC determination of whether discrimination was a circumstance of the death.^5^ Analysis of the discrimination circumstance was restricted to deaths reviewed by MMRCs after May 29, 2020. These circumstances are defined as whether obesity/substance use disorder/mental health condition/discrimination contributed to the death, and not just whether the circumstance was present.

A death is considered preventable if the MMRC determines there was at least some chance of the death being averted by one or more reasonable changes to patient, family, provider, facility, systems factors, and/or community.^3^ If a death is determined to be preventable, the MMRCs describe, using free text, contributing factors, and recommendations among pregnancy-related deaths. For each contributing factor described, MMRCs select a contributing factor class from a standardized list of 27 specific contributing factor classes. Each preventable pregnancy-related death can have multiple contributing factors and classes. MMRCs also make recommendations for preventing future pregnancy-related deaths for each contributing factor they identify.

For this report, we reviewed all MMRC recommendations among the 5 most common contributing factor classes. We selected example MMRC recommendations to represent each of the 5 most frequent contributing factor classes. MMRC recommendations included in this report may have been edited slightly for clarity.

### Statistical analysis

Descriptive statistics were calculated as counts and percentages. Missing values are reported but not included in calculations of distributions. All analyses were performed using SAS, version 9.4 (SAS Institute Inc).

## Results

Overall, there were 210 pregnancy-related deaths, which occurred in 2017 to 2019 among residents of the 32 states, with an MMRC-determined underlying cause of death attributed to CVs. This includes 84 (40.0%) pregnancy-related deaths with a CM as the underlying cause of death and 126 (60.0%) pregnancy-related deaths with an OCV as the underlying cause of death.

Demographic characteristics of the CV, CM, and OCV deaths are shown in Table 1. More than half of CM deaths (51.2%) were among non-Hispanic Black persons. Non-Hispanic Black and White persons each represented about 40% of OCV deaths. Among CV deaths overall, two-thirds (66%) occurred among people under the age of 35 years and 60% were among people with a high school education or less. Only 58% of the pregnancy-related CV deaths had an autopsy performed (Table 1).

**Table 1:** Characteristics of Pregnancy-Related Total Cardiovascular Conditions, Cardiomyopathy, and Other Cardiovascular Conditions Deaths^a^

	Total Cardiovascular Conditions (N = 210)	Cardiomyopathy (n = 84)	Other Cardiovascular Conditions (n = 126)
Race and ethnicity			
Hispanic	20 (9.8)	5 (6.1)	15 (12.2)
Non-Hispanic AI/AN	1 (0.5)	0 (0.0)	1 (0.8)
Non-Hispanic Asian	9 (4.4)	2 (2.4)	7 (5.7)
Non-Hispanic Black	90 (43.9)	42 (51.2)	48 (39.0)
Non-Hispanic NHOPI	0 (0.0)	0 (0.0)	0 (0.0)
Non-Hispanic White	82 (40.0)	33 (40.2)	49 (39.8)
Non-Hispanic all other/multiple races	3 (1.5)	0 (0.0)	3 (2.4)
Missing	5	2	3
Age (y)			
15-19	8 (3.8)	3 (3.6)	5 (4.0)
20-24	25 (12.0)	11 (13.1)	14 (11.2)
25-29	43 (20.6)	21 (25.0)	22 (17.6)
30-34	61 (29.2)	19 (22.6)	42 (33.6)
35-39	54 (25.8)	22 (26.2)	32 (25.6)
40-44	15 (7.2)	7 (8.3)	8 (6.4)
45+	3 (1.4)	1 (1.2)	2 (1.6)
Missing	1	0	1
Education level			
12th grade or less; no diploma	34 (16.6)	11 (13.6)	23 (18.5)
High school grade or GED	89 (43.4)	34 (42.0)	55 (44.4)
Some college credit; no degree	38 (18.5)	17 (21.0)	21 (16.9)
Associate or bachelor’s degree	34 (16.6)	15 (18.5)	19 (15.3)
Advanced degree	10 (4.9)	4 (4.9)	6 (4.8)
Missing	5	3	2
Was there an autopsy?			
Yes	120 (58.3)	43 (51.8)	77 (62.6)
Report available	120	43	77
Report not available	0	0	0
No	86 (41.8)	40 (48.2)	46 (37.4)
Missing	4	1	3

Values are n (%).

AI/AN = American Indian or Alaska Native; GED = general education diploma; NHOPI = Native Hawaiian or other Pacific Islander.

a Excludes hypertensive disorders of pregnancy and cerebrovascular accidents. Data with missing values are not included in the distribution percentages.

**Figure 1:**
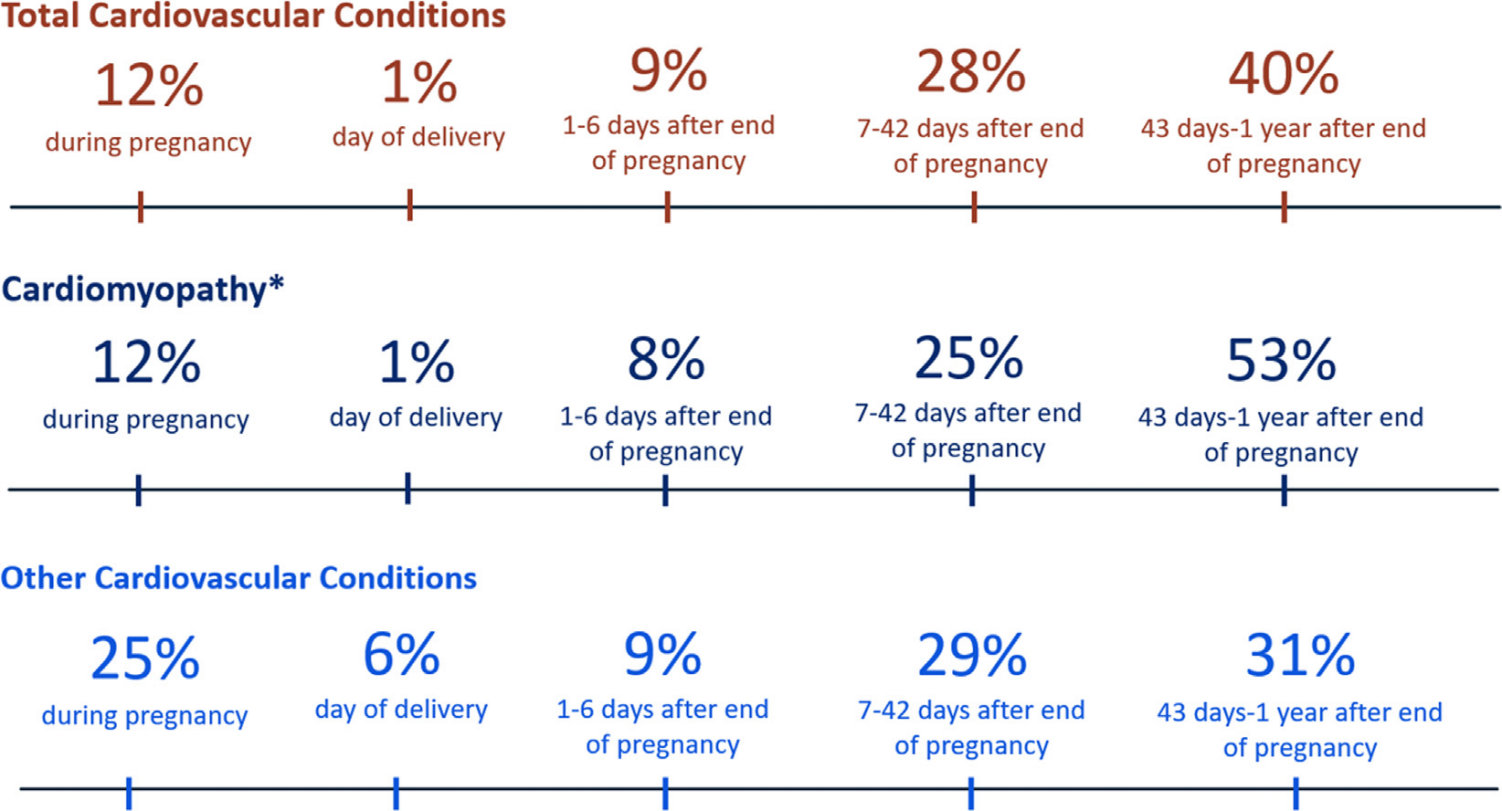
**Timing of Preventable Pregnancy-Related Cardiovascular Deaths** Timing of death was missing or unknown for 1 pregnancy-related cardiomyopathy death. The figure shows the percentage of pregnancy-related deaths with an MMRC-identified underlying cause of death of cardiovascular conditions (excluding hypertensive disorders of pregnancy and cerebrovascular accidents) at 5 time periods from pregnancy to 1-year postpartum. The percentages are displayed for total cardiovascular conditions, and then for cardiomyopathy and other cardiovascular conditions individually. Percentages might not sum to 100 because of rounding. MMRC = Maternal Mortality Review Committee.

Pregnancy-related CV deaths with information on timing of death are presented overall and by CM and OCV in Figure 1. Among CV deaths overall, two-thirds (67%) occurred from 7 days to 1 year following the end of pregnancy, including 40% who died from 43 days to 1 year after the end of pregnancy. Among the pregnancy-related CM deaths, over half (53%) occurred 43 days to 1 year after the end of pregnancy, while 12% occurred during pregnancy. Among pregnancy-related OCV deaths, 31% occurred 43 days to 1 year after the end of pregnancy, while 25% occurred during pregnancy.

The distribution of the specific CV-related MMRC-identified underlying cause of death subcategories is presented in Table 2. More than half (56.0%) of all pregnancy-related CM deaths were attributed to postpartum/peripartum CM, and 7.1% attributed to hypertrophic CM. Almost two-thirds (64.3%) of CM deaths among non-Hispanic Black persons were due to postpartum/peripartum CM, and 2.4% were due to hypertrophic CM (data not shown). In contrast, about half (51.5%) of the CM deaths among non-Hispanic White persons were due to postpartum/peripartum CM, and 9.1% were due to hypertrophic CM (data not shown). Among the pregnancy-related deaths due to OCV, the most frequent subcategories of cause of death were vascular aneurysm/dissection (19.8%), hypertensive cardiovascular disease (14.3%), coronary artery disease/myocardial infarction/atherosclerotic cardiovascular disease (11.1%), conduction defects/arrhythmias (11.1%), and valvular heart disease (8.7%) (Table 2).

**Table 2:** Specific Cause of Death Subcategories Among Cardiomyopathy and Other Cardiovascular Conditions Pregnancy-Related Deaths^a^

	Cardiomyopathy (n = 84)	Other Cardiovascular Conditions (n = 126)
Postpartum/peripartum cardiomyopathy	47 (56.0)	- (–)
Hypertrophic cardiomyopathy	6 (7.1)	- (–)
Other cardiomyopathy/NOS	31 (36.9)	- (–)
Coronary artery disease/myocardial infarction/atherosclerotic cardiovascular disease	- (–)	14 (11.1)
Pulmonary hypertension	- (–)	6 (4.8)
Valvular heart disease	- (–)	11 (8.7)
Vascular aneurysm/dissection	- (–)	25 (19.8)
Hypertensive cardiovascular disease	- (–)	18 (14.3)
Marfan syndrome	- (–)	2 (1.6)
Conduction defects/arrhythmias	- (–)	14 (11.1)
Vascular malformations outside head and coronary arteries	- (–)	1 (0.8)
Cardiovascular/NOS, including congestive heart failure, cardiomegaly, cardiac hypertrophy, cardiac fibrosis, nonacute myocarditis	- (–)	35 (27.8)

Values are n (%).

NOS = not otherwise specified.

a Excludes hypertensive disorders of pregnancy and cerebrovascular accidents.

A preventability determination was made by the MMRCs for 97.6% of pregnancy-related CV deaths. Overall, 76.1% of CV deaths were determined to be preventable, with 77.1% of CM and 75.4% of OCV deaths identified by MMRCs as having at least some chance of prevention (Table 3).

**Table 3:** Preventability and Circumstances Surrounding Death Among Total Cardiovascular Conditions, Cardiomyopathy, and Other Cardiovascular Conditions Deaths^a^

	Total Cardiovascular Conditions (N = 210)	Cardiomyopathy (n = 84)	Other Cardiovascular Conditions (n = 126)
Was the death preventable?			
Yes	156 (76.1)	64 (77.1)	92 (75.4)
No	49 (23.9)	19 (22.9)	30 (24.6)
Missing or unable to determine	5	1	4
Was obesity a circumstance of the death?			
Yes	68 (32.4)	28 (33.3)	40 (31.7)
Probably	29 (13.8)	12 (14.3)	17 (13.5)
No	105 (50.0)	39 (46.4)	66 (52.4)
Unknown	8 (3.8)	5 (6.0)	3 (2.4)
Was substance use disorder a circumstance of the death?			
Yes	23 (11.1)	14 (16.7)	9 (7.3)
Probably	11 (5.3)	3 (3.6)	8 (6.5)
No	160 (76.9)	59 (70.2)	101 (81.5)
Unknown	14 (6.7)	8 (9.5)	6 (4.8)
Missing	2		2
Were mental health conditions a circumstance of the death?			
Yes	17 (8.2)	6 (7.1)	11 (8.9)
Probably	14 (6.7)	6 (7.1)	8 (6.5)
No	158 (76.0)	63 (75.0)	95 (76.6)
Unknown	19 (9.1)	9 (10.7)	10 (8.1)
Missing	2		2
Was discrimination a circumstance of the death?			
Yes	14 (12.2)	5 (12.5)	9 (12.0)
Probably	17 (14.8)	5 (12.5)	12 (16.0)
No	51 (44.3)	15 (37.5)	36 (48.0)
Unknown	33 (28.7)	15 (37.5)	18 (24.0)
Missing	11	7	4

Values are n (%). Circumstances are defined as whether the condition/circumstance contributed to the death, and not just whether it was present.

Discrimination assessed among 126 pregnancy-related deaths (CM = 47, OCV = 79) reviewed after this question was added to MMRIA on May 29, 2020.

Data missing values are not included in the distribution percentages.

a Excludes hypertensive disorders of pregnancy and cerebrovascular accidents.

MMRC determinations of circumstances surrounding CV deaths are shown in Table 3. MMRCs identified that obesity was a circumstance (yes or probably) in almost half of CM deaths (47.6%) and OCV deaths (45.2%). There were 115 pregnancy-related CV deaths reviewed on or after May 29, 2020, which included an MMRC determination for whether discrimination was a circumstance of the death. MMRCs identified that discrimination was a circumstance (yes or probably) in 25.0% of the CM deaths and 28.0% of the OCV deaths (Table 3).

A total of 944 contributing factor classes were reported by MMRCs, for a mean of approximately 6 contributing factor classes for each preventable pregnancy-related CV death (Table 4). The most commonly identified contributing factor classes were Knowledge (n = 115, 12.2%), Clinical Skill/Quality of Care (n = 111, 11.8%), Continuity of Care/Care Coordination (n = 103, 10.9%), Chronic Disease (n = 79, 8.4%), and Access/Financial (n = 64, 6.8%). These 5 contributing factor classes accounted for 50% of the total identified by MMRCs.

**Table 4:** Contributing Factor Classes Among Preventable Pregnancy-Related Total Cardiovascular Conditions, Cardiomyopathy, and Other Cardiovascular Conditions Deaths^a^

	Total Cardiovascular Conditions (N = 944)	Cardiomyopathy (n = 402)	Other Cardiovascular Conditions (n = 542)
Knowledge	115 (12.2	62 (15.4	53 (9.8)
Clinical Skill/Quality of Care	111 (11.8)	47 (11.7)	64 (11.8)
Continuity of Care/Care Coordination	103 (10.9)	33 (8.2)	70 (12.9)
Chronic Disease	79 (8.4)	28 (7.0)	51 (9.4)
Access/Financial	64 (6.8)	25 (6.2)	39 (7.2)
Assessment	56 (5.9)	13 (3.2)	43 (7.9)
Delay	56 (5.9)	30 (7.5)	26 (4.8)
Communication	48 (5.1)	26 (6.5)	22 (4.1)
Adherence	45 (4.8)	18 (4.5)	27 (5.0)
Policies/procedures	42 (4.5)	17 (4.2)	25 (4.6)
Substance use disorder	37 (3.9)	14 (3.5)	23 (4.2)
Discrimination	35 (3.7)	12 (3.0)	23 (4.2)
Referral	33 (3.5)	17 (4.2)	16 (3.0)
Social support/isolation	23 (2.4)	13 (3.2)	10 (1.8)
Other	18 (1.9)	8 (2.0)	10 (1.8)
Tobacco use	14 (1.5)	5 (1.2)	9 (1.7)
Mental health conditions	13 (1.4)	8 (2.0)	5 (0.9)
Outreach	12 (1.3)	7 (1.7)	5 (0.9)
Structural racism	10 (1.1)	4 (1.0)	6 (1.1)
Cultural/religious	7 (0.7)	3 (0.7)	4 (0.7)
Equipment/technology	6 (0.6)	5 (1.2)	1 (0.2)
Violence	5 (0.5)	3 (0.7)	2 (0.4)
Environmental	4 (0.4)	1 (0.2)	3 (0.6)
Unstable housing	3 (0.3)	2 (0.5)	1 (0.2)
Interpersonal racism	2 (0.2)	0 (0.0)	2 (0.4)
Legal	1 (0.1)	0 (0.0)	1 (0.2)
Personnel	1 (0.1)	0 (0.0)	1 (0.2)
Trauma	1 (0.1)	1 (0.2	0 (0.0)

Values are n (%). Complete list of all 27 specific contributing factor classes, and “other”, available for selection by Maternal Mortality Review Committees.

1 preventable pregnancy-related death had no contributing factor classes specified.

a Excludes hypertensive disorders of pregnancy and cerebrovascular accidents.

### Example MMRC recommendations

Example MMRC recommendations within the 5 most frequent contributing factor classes are listed in Table 5. Knowledge was the most frequent contributing factor class. Example recommendations that address Knowledge included: “community-based organizations should develop public education campaigns to raise awareness of warnings signs of early postpartum complications, when to seek care, and emphasize 1-year postpartum as critical window"; and “Care Coordination or Navigators at the managed care organization level and community support services to assess where patient is at, what they understand, and any needs they have” (Table 5).

**Table 5:** Example MMRC Recommendations Addressing Most Frequent Contributing Factor Classes Among Preventable Pregnancy-Related Cardiovascular Conditions Deaths^a^^,^^b^

Contributing Factor Class	Example MMRC Recommendations
Knowledge	Community-based organizations should develop public education campaigns to raise awareness of warnings signs of early postpartum complications, explain when to seek care, and emphasize 1-y postpartum as critical window.
	Providers should assure that there are appropriate instructions, given at the time of discharge, for when to call with problems.
	Providers who treat pregnant and postpartum women should adhere to evidence-based guidelines and practices to support high-quality care of maternal hypertension and should ensure appropriate consultation and referral practices.
	All delivering facilities should provide education regarding postpartum warning signs prior to discharge.
	Care Coordination or Navigators at the managed care organization level and community support services to assess: where patient is at, what they understand, and any needs they have. Universal access to community health workers for all expectant families.
Clinical Skill/Quality of Care	All providers should educate themselves regarding the American College of Obstetricians and Gynecologists (ACOG) guidelines for screening for cardiovascular disease in pregnancy.
	Providers and managed care organizations should ensure that their patients (members) with prior cardiac history have consultation with cardiologist (preconception, prenatally, and postpartum) and that there are processes in place to ensure coordination of care and information sharing as soon as possible.
	Facilities and providers should complete comprehensive postpartum discharge planning for high-risk pregnancies/deliveries in alignment with ACOG Committee Opinion #736: Optimizing Postpartum Care.
	Hospital emergency departments should have policies for identifying pregnancy/postpartum status for all women who present for care.
	Professional societies should provide education to providers on signs and symptoms of aortic dissection.
	ACOG and partners should develop an emergency room bundle for the care of pregnant women.
Continuity of Care/Care Coordination	Patients should be able to have access to continued care with one provider house (care umbrella with one provider/clinic in the lead) throughout the course of a person's illness/care.
	A patient who is identified as high risk needs a care coordinator who is following her individually, throughout the course of care, to assure she goes back to the same care provider each time and helps her navigate information and the system. This person would also coordinate the patient’s appointments, help her with mental health referrals, and encourage compliance with medical recommendations.
	Hospitals should assign designated care coordinators to patients requiring multidisciplinary care at first contact with health care system after emergency department visit.
	Facilities should have a system to follow-up with patients discharged after preeclampsia/hemorrhage diagnoses and make sure patients see follow-up providers, including cardiology.
	Hospital systems should have patient-centered, multidisciplinary, coordinated care (with use of bundles) as part of discharge planning for patients with chronic illnesses, especially during the postpartum period.
	Advocate for system integration for inpatient-to-outpatient transitions and specialist-to-primary care transitions.
Chronic Disease	Providers should refer all patients with history of complex medical issues to case management services during the first prenatal visit and throughout the pregnancy and postpartum.
	Providers should provide reproductive life planning/interconception care/family planning counseling to women with chronic conditions.
	Providers should educate women on the importance of preconception health, especially in the context of chronic disease and/or obesity.
	Obstetric providers should refer patients with reported cardiac conditions to a cardiologist during pregnancy and postpartum.
	Facilities and payers should provide case management services to women with chronic health conditions during pregnancy and postpartum.
	Community-based organizations should educate women on the importance of preconception health, especially in the context of chronic disease and/or obesity.
Access/Financial	Hospitals should employ a social worker or case manager who can conduct and document a psychosocial needs assessment prior to delivery hospital discharge—to identify potential barriers to care and connect women to resources and postpartum case management.
	Insurers should provide navigation services to individuals with transportation barriers—to use transportation as a covered benefit.
	Medicaid should improve the application process to make it user friendly and more easily accessible.
	Medicaid should streamline enrollment to support early and easy entry into prenatal care (including specialty care).
	Medicaid should be extended to 1 year for all postpartum women, particularly for those with hypertensive disorders of pregnancy; and enrollment and maintenance processes should be streamlined.

MMRC = Maternal Mortality Review Committee.

a Maternal Mortality Review Committee recommendations were edited slightly for clarity.

b Excludes hypertensive disorders of pregnancy and cerebrovascular accidents.

Clinical Skill/Quality of Care was the second most frequent contributing factor class. Examples of MMRC recommendations addressing this class included: “providers and managed care organizations should ensure that their patients and members have consultation with cardiologist for people (preconception, prenatally, and postpartum) with prior cardiac history and that there are processes in place to ensure coordination of care and information sharing as soon as possible”; and “professional societies should provide education to providers on signs and symptoms of aortic dissection” (Table 5).

Continuity of Care/Care Coordination was third most frequent contributing factor class. Examples of MMRC recommendations that address this contributing factor included: “patient who is identified as high risk needs a care coordinator who is following her individually, throughout the course of care, to assure she goes back to the same care provider each time and helps her to navigate information and the system. This person would also coordinate her appointments, help her with mental health referrals, and encourage compliance with medical recommendations”; and “advocate for system integration for inpatient to outpatient and specialist to primary care” (Table 5).

Chronic Disease was fourth most frequent contributing factor class. Examples of MMRC recommendations that address Chronic Disease included examples such as: “community-based organizations should educate women on the importance of preconception and interconception health, especially in the context of chronic disease and/or obesity”; and “facilities and payers should provide case management services to women with chronic health conditions during pregnancy and postpartum” (Table 5).

Access/Financial was the fifth most frequent contributing factor class. Examples of MMRC recommendations that address these contributing factors include “hospitals should employ a social worker or case manager who can conduct and document a psychosocial needs assessment prior to delivery hospital discharge to identify potential barriers to care and connect women to resources and postpartum case management”; and “Medicaid should improve the application process to make it user friendly and more easily accessible” (Table 5).

## Discussion

MMRCs determined that 76% of CV deaths were preventable. The most common contributing factor classes were Knowledge, Clinical Skill/Quality of Care, and Continuity of Care/Care Coordination (Central Illustration). The detailed multidisciplinary MMRC reviews, based on the context of their state, position committees to develop specific and actionable recommendations. The breadth of the 5 most frequent contributing factors and the average number (6) associated with each pregnancy-related death are emblematic of the complexity of pregnancy-related mortality. The social ecological model provides a framework for considering the complex interplay among multilevel contexts.^6^ Committees consider not only the health care and clinical events near the time of death; they also can include the broader context using tools to understand the social and built environments, neighborhood resources, and structural inequality.^7^ The social ecological model stresses that these multiple contexts are interrelated, highlighting the importance of addressing multiple dimensions simultaneously to improve health outcomes.^6^

**Central Illustration:**
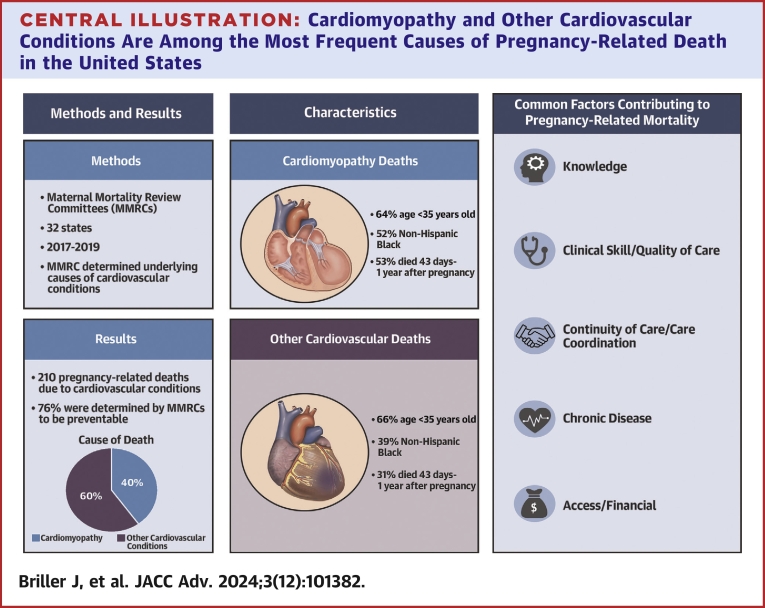
**Cardiomyopathy and Other Cardiovascular Conditions Are Among the Most Frequent Causes of Pregnancy-Related Death in the United States** We analyzed pregnancy-related death data from MMRCs in 32 states, occurring during 2017 to 2019, with MMRC-determined underlying causes of cardiovascular conditions. Over 75% of pregnancy-related deaths were determined by MMRCs to be preventable. The five most common contributing factor classes were knowledge, clinical skill/quality of care, continuity of care/care coordination, chronic disease, access/financial. Example MMRC recommendations illustrate prevention opportunities that address contributing factors, including broader awareness of urgent warning signs, improved handoffs for care coordination and continuity, and expanded accessibility of community-based comprehensive and integrated care services. Abbreviation as in Figure 1.

Knowledge was the most commonly identified contributing factor class among CV deaths. Example MMRC recommendations, intended to prevent future deaths from this contributing factor class, included increasing knowledge about urgent maternal warning signs. CDC’s Division of Reproductive Health’s *Hear Her* campaign seeks to raise awareness of urgent maternal warning signs during and after pregnancy and improve communication between patients and their health care providers.^8^ Both patient-facing and provider-facing materials are available. Health system support for this awareness can include facility-based programs that educate nurses and other health care professionals about the timeline for postpartum risk and the significance of postpartum maternal mortality.^9^

Over half (53%) of CM deaths and almost one-third (31%) of deaths from OCV occurred in the late postpartum period (43 days to 1 year after the end of pregnancy). The timing of these deaths emphasizes the need for tailored postpartum care and a multidisciplinary approach. The creation and use of comprehensive postpartum plans—with careful consideration of each patient’s risk profile and access to resources—has been proposed to facilitate effective handoffs needed in the first few days to months after pregnancy ends.^10^^,^^11^ The American College of Obstetricians and Gynecologists (ACOG) recommends a postpartum follow-up visit with either the primary care provider or cardiologist within 7 to 14 days of delivery for women with heart disease/cardiovascular disorders.^12^ Individuals identified as high risk should be evaluated at 3 months in a comprehensive cardiovascular postpartum visit with a Pregnancy Heart Team or cardio-obstetrics team, obstetrician-gynecologist, or other primary care provider.^11^^,^^12^ Women with cardiovascular risk factors or new-onset CV may be unable to access ongoing treatment because of gaps in health care coverage in the postpartum period.^13^^,^^14^

Example MMRC recommendations related to the contributing factor class of Access/Financial include extension of Medicaid for 12 months postpartum along with simplification of the enrollment and maintenance process for Medicaid. As of February 2024, 45 states have implemented a 12-month postpartum extension of Medicaid.^15^ In addition, Medicaid reforms to reduce burdens to care—including continuous eligibility, presumptive eligibility, and coordinated care services—may enable more consistent access to care, including prenatal care.^16^

Example MMRC recommendations related to the contributing factor class of Continuity of Care/Care Coordination identified the need to address gaps in care coordination and transition of care between prenatal care, specialty care, hospitalization, postpartum care, and ongoing health maintenance. Integrated patient care includes coordination of care both within and across teams and with the community, along with patient-centeredness and shared responsibility.^17^ A recent systematic review of care coordination programs in pregnancy noted that “although the components of the care coordination programs included (in the analysis) suggest only a modest improvement in fetal outcomes, the benefits to the participants—both patients and providers—may extend beyond the brief course of the pregnancy.”^18^ Additional research, to evaluate the impact of integrated care in pregnancies with complications on broader, long-term outcomes, could be beneficial.

Example MMRC recommendations related to the contributing factor class of Clinical Skills included screening, risk identification, and consultation or referral of people identified as high risk. Screening algorithms have been recommended by ACOG^12^ and others,^19^^,^^20^ and screening for CV conditions in pregnancy has been recommended as a quality measure.^21^ Consultation with or referral to a cardio-obstetrics team (also known as Pregnancy Heart Team) for risk stratification and multidisciplinary care is recommended^12^^,^^22^ because late pregnancy assessment has been associated with frequent adverse cardiac complications during pregnancy.^2^^,^^23^ Multidisciplinary meetings of the cardio-obstetrics team facilitate patient-centered coordination of planning antenatal, delivery, and postpartum care.^12^^,^^22^

Example MMRC recommendations related to the contributing factor class of Chronic Disease include the role of preconception health optimization. Recent studies have evaluated the prevalence of cardiovascular risk factors (including hypertension, diabetes, obesity, and tobacco use) among persons of reproductive age (age 20-44 years).^24^, ^25^, ^26^ Prevalence rates for hypertension and diabetes were higher among non-Hispanic Black individuals than Hispanic or non-Hispanic White individuals.^25^^,^^26^ The importance of preconception health is consistent with the need to move upstream to address the prevalence of these conditions and address the gap in cardiovascular disease prevention in younger adults.^27^

Two-thirds (66%) of the pregnancy-related deaths due to CV occurred among people under the age of 35 years, as did the majority of all pregnancy-related deaths and the majority of births.^3^ Women aged 18 to 55 years are 50% more likely than similar aged men to present without chest pain when they have ST-segment elevation myocardial infarction, with 1 in 5 women perceiving their symptoms as related to anxiety or stress vs acute myocardial infarction.^28^, ^29^, ^30^ Spontaneous coronary artery dissection causes more than 40% of myocardial infarctions in pregnancy and the postpartum period.^30^ spontaneous coronary artery dissection, which has been clinically underrecognized, occurs predominantly in young women (mean age 40-42 years) with few or no conventional risk factors for atherosclerosis.^31^^,^^32^

Vascular aneurysms/dissections accounted for 19.8% of the deaths due to OCV. A recent analysis of aortic dissection during pregnancy identified Marfan syndrome, primary hypertension, and preeclampsia/eclampsia as significantly associated with the risk of aortic dissection during pregnancy and the puerperium.^33^ There is a growing need to identify high-risk patients and provide them with aggressive prevention and monitoring.^33^, ^34^, ^35^ Efforts at the health system level are required to increase assess to perinatal care because earlier interventions may result in more favorable outcomes.^33^ Prompt diagnosis and therapy are noted by some to be the only factors critical to acute aortic dissection survival.^35^

Example MMRC recommendations for the contributing factor class of Clinical Skill/Quality of Care noted the need for education to enhance recognition and management of these CV conditions among clinicians, including those in emergency medicine and obstetrician-gynecologists. In response to MMRC findings and recommendations for enhanced emergency care, ACOG recently developed and released resources to help practitioners in nonobstetric settings identify and manage pregnancy-related emergencies, including cardiovascular disease in pregnancy and postpartum.^36^

The strengths of our analysis include the detailed data available from MMRCs in 32 states. The committees in these states use a broad array of data sources and multidisciplinary memberships to provide a deeper understanding of pregnancy-related mortality—recognizing medical and nonmedical contributors to deaths. The identified contributing factor classes and example MMRC recommendations highlight options to prioritize interventions for reducing pregnancy-related deaths due to CV conditions.

### Study limitations

Despite the strengths of this multistate analysis, there are limitations. The data are aggregated from individual state-based review committees. While MMRCs utilize a standardized review process, variations in review may have existed. MMRIA data are based on the availability and completeness of abstracted data. Not every state contributed data, and partial years of death were included; thus, findings are not representative of all pregnancy-related CV deaths nor does this represent a population-based census of pregnancy-related deaths due to CV. Cause-specific pregnancy-related mortality ratios cannot be calculated. While most CM deaths were attributed to postpartum and peripartum CM, a large percentage were classified as other CM/NOS. Similarly, a significant proportion of the deaths due to OCV were categorized as other/NOS and may belong in a more specific condition category. The contributing factor classes of discrimination, interpersonal racism, and structural racism were not added to the MMRIA Committee Decisions Form until May 2020 and would not have been considered in the MMRC review of all pregnancy-related deaths in this analysis. Therefore, these classes may have contributed to pregnancy-related deaths more frequently than reported in this analysis. We present example MMRC recommendations among only the 5 most frequently occurring contributing factor classes; thus, this does not represent the full spectrum of the MMRC-identified prevention opportunities. Despite these limitations, this study adds to our understanding of CV as a major contributor to pregnancy-related mortality.

## Conclusions

Most pregnancy-related deaths due to CV are preventable, and recommendations for preventing future deaths and improving maternal outcomes require interventions across multiple contexts. Jurisdiction-based MMRCs evaluate and interpret comprehensive data and provide information on specific contributing factor classes and recommendations in the local context. Common contributing factor classes and example MMRC recommendations provided in this report illustrate the breadth of prevention opportunities—such as broader awareness of urgent warning signs, improved handoffs for care coordination and continuity, and expanded accessibility of community-based comprehensive and integrated care services.

Perspectives **COMPETENCY IN MEDICAL KNOWLEDGE:** CV are the leading cause of pregnancy-related death among non-Hispanic Black persons. Pregnancy-related deaths due to CV occurred during pregnancy, delivery, and in the year following the end of pregnancy; the majority of deaths were due to acquired conditions and among persons ≤35 years of age.

**COMPETENCY IN PATIENT CARE AND PROCEDURAL SKILLS:** Clinicians’ and patients’ improved awareness of signs and symptoms of CV were common recommendations by MMRCs to prevent similar deaths. Coordination of care transitions between prenatal care, specialty care, hospitalization, postpartum care, and ongoing health maintenance were also common recommendations for prevention.

**TRANSLATIONAL OUTLOOK:** MMRCs provide detailed reviews of pregnancy-related deaths. Addressing the identified contributing factor classes and recommendations for prevention may reduce pregnancy-related mortality among disproportionately impacted populations.

## Funding support and author disclosures

This project was supported in part by an appointment to the Research Participation Program at the Centers for Disease Control and Prevention. There is no additional financial support received for the research, authorship, and/or publication of this article. The authors have reported that they have no relationships relevant to the contents of this paper to disclose.
