# Supplementary material for: Pregnancy-Related Mortality Due to Cardiovascular Conditions: Maternal Mortality Review Committees in 32 U.S. States, 2017 to 2019
Source: JACC Adv. 2024 Nov 8;3(12):101382. doi: 10.1016/j.jacadv.2024.101382 (PMC11585746; doi:10.1016/j.jacadv.2024.101382)
Supplement: Supplemental material [file mmc1.docx]

**Supplemental Appendix**

States with pregnancy-related deaths due to Cardiovascular Conditions, MMRIA, 2017-2019

Alaska, Arkansas, Arizona, California, Colorado, Connecticut, Delaware, Florida, Georgia, Hawaii, Illinois, Indiana, Kansas, Louisiana, Massachusetts, Minnesota, Missouri, Mississippi, North Carolina, New Hampshire, New Jersey, New Mexico, New York, Ohio, Pennsylvania, Tennessee, Texas, Washington, Wisconsin, West Virginia, Wyoming
